# Evidences for redox reaction driven charge transfer and mass transport in metal-assisted chemical etching of silicon

**DOI:** 10.1038/srep36582

**Published:** 2016-11-08

**Authors:** Lingyu Kong, Binayak Dasgupta, Yi Ren, Parsian K. Mohseni, Minghui Hong, Xiuling Li, Wai Kin Chim, Sing Yang Chiam

**Affiliations:** 1 NUS Graduate School for Integrative Sciences and Engineering, National University of Singapore, 28 Medical Drive, 117456, Singapore; 2 Institute of Materials Research and Engineering, A*STAR (Agency for Science, Technology and Research), 2 Fusionopolis Way, Innovis, 138634, Singapore; 3 Department of Electrical and Computer Engineering, National University of Singapore, 4 Engineering Drive 3, 117583, Singapore; 4 Department of Electrical and Computer Engineering, Micro and Nanotechnology Laboratory, University of Illinois at Urbana-Champaign, Urbana, Illinois 61801, United States

## Abstract

In this work, we investigate the transport processes governing the metal-assisted chemical etching (MacEtch) of silicon (Si). We show that in the oxidation of Si during the MacEtch process, the transport of the hole charges can be accomplished by the diffusion of metal ions. The oxidation of Si is subsequently governed by a redox reaction between the ions and Si. This represents a fundamentally different proposition in MacEtch whereby such transport is understood to occur through hole carrier conduction followed by hole injection into (or electron extraction from) Si. Consistent with the ion transport model introduced, we showed the possibility in the dynamic redistribution of the metal atoms that resulted in the formation of pores/cracks for catalyst thin films that are ≲30 nm thick. As such, the transport of the reagents and by-products are accomplished via these pores/cracks for the thin catalyst films. For thicker films, we show a saturation in the etch rate demonstrating a transport process that is dominated by diffusion via metal/Si boundaries. The new understanding in transport processes described in this work reconcile competing models in reagents/by-products transport, and also solution ions and thin film etching, which can form the foundation of future studies in the MacEtch process.

Silicon (Si) nanostructures are important building blocks for many technological applications. In particular, silicon nanowires (NWs) find a wide array of usage in areas such as electronics^1^,^2^, photovoltaics^3^,^4^, energy storage^5^, optical devices^6^,^7^, catalysis^8^, drug delivery^9^, thermoelectrics^10^, as well as biological and chemical sensors^11^,^12^,^13^. The importance of fabricating reliable Si nanowires has provided the motivation for the exploration of many different fabrication methods. These include vapour-liquid-solid growth, molecular beam epitaxy, and reactive ion etching. Of the available techniques, metal-assisted chemical etching (MacEtch) remains a key approach in achieving large area and low temperature fabrication at relatively low cost. MacEtch has also the added advantage of achieving ordered crystalline Si nanostructures without damages, as opposed to those prepared using high-energy ions in reactive ion etching. As such, various reports have utilized MacEtch to yield the fabrication of well-defined and high quality semiconductor nanostructures, such as nanowires, nanoplates or nanofins and bulk microstructures^14^,^15^,^16^,^17^,^18^,^19^.

There has been a great deal of progress made in understanding the MacEtch process and this is well summarized in a few reviews^20^,^21^. Here, we briefly describe the key steps in the currently accepted MacEtch mechanism. The metal catalyst (e.g., Au or Ag) is the key ingredient that accelerates the etching of Si. This is achieved by accelerating the generation of hole charges when hydrogen peroxide (H_2_O_2_) is reduced at the electrolyte-metal catalyst interface, according to the following reaction:


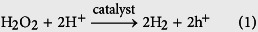


The hole charges, h^+^, are then transported to the metal/silicon interface whereby oxidation of Si occurs either via hole injection into Si or electron extraction from Si. The presence of the generated holes speeds up the dissolution of Si via the following reaction:


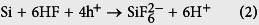


This results in accelerated etching for those Si regions that are in contact with the metal catalyst. The nature of the method allows for the creation of desired structures simply by patterning the metal catalyst on the Si substrate^20^,^21^. For example, uniform silicon nanowires can be fabricated by MacEtch using mesh-patterned metal catalyst films on Si.

While the above-mentioned model is generally well accepted, there are still a few key phenomena that lacked coherent explanations. In particular, there is still a lack of understanding in the fundamental reasons behind the stop-etch phenomenon shown by metals like chromium (Cr)^22^,^23^,^24^,^25^, and also debates about the mass transport during etching^4^,^15^,^18^,^20^,^23^,^26^,^27^,^28^. This knowledge is important to understand the basic MacEtch mechanism. Thereafter, this knowledge can enhance the versatility of design by providing choices in the selection of blocking metals or metal catalysts. In this work, we will focus on three key aspects of MacEtch related to transport/transfer processes. The first is the mass transport of the chemical species involved in the etching. The second is the transport process of hole charges to Si and finally, the oxidative charge transfer process at the metal/Si interface. We provide clear evidence and understanding on the transport pathway of the reagents and by-products, which have previously been elusive. We also show that the charge injection process at the metal/Si interface can be governed by a reaction driven transfer. Finally, we demonstrate that the charge transport process to the Si interface can be accomplished by ion transport, instead of the widely accepted electronic hole conduction. The ion transport process provides a more unified picture for MacEtch and helps to explain several phenomena: (1) Creation of porous layer in the metal catalyst, (2) failure of both work function and electronegativity in governing the charge transfer, and (3) reconciliation of metal ions etching and thin film etching.

## Results and Discussion

**Figure 1:**
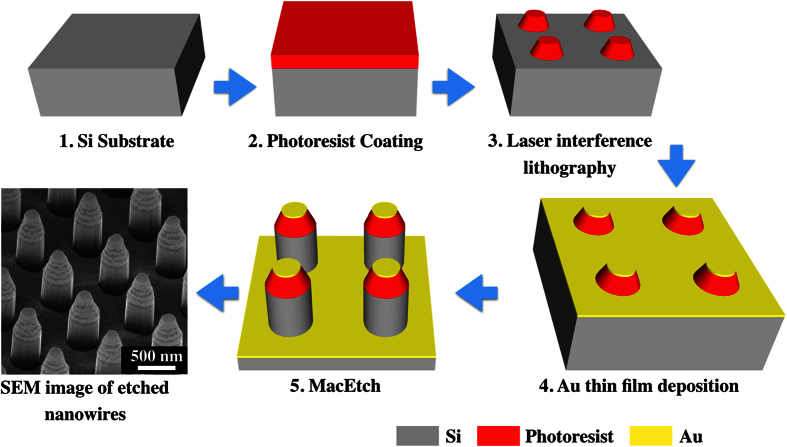
Schematic showing the process flow in the fabrication of silicon nanowires by laser interference lithography. The final image shows a high-resolution SEM micrograph of etched nanowires.

Two different patterning methods were used, namely laser interference lithography patterned Au metal mesh and shadow masking patterned Au circular dots. The former technique is able to provide nanostructures (i.e., Si nanowires array) with similar diameter and spacing to ensure that the etching results are consistent. The schematic illustration of the fabrication steps by this approach is shown in Fig. 1. The shadow masking patterned Au dots were used for X-ray photoelectron spectroscopy (XPS) analysis, the large pattern size ensures that the etched area is bigger than the X-ray spot size. Samples were prepared on Si substrates and the catalytic etching was conducted in a solution consisting of 4.6 M HF, 0.15 M H_2_O_2_ and deionized water. All etched samples are imaged by high-resolution field emission Scanning Electron Microscopy (SEM). Chemical analysis was achieved using a XPS system. The obtained results are analyzed for MacEtch mass and charge transport study.

### Transport of reagents and by-products in etching

**Figure 2:**
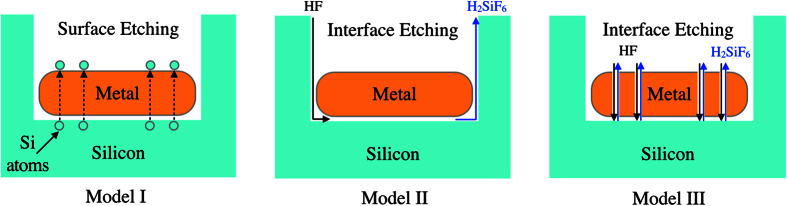
Schematic illustration of three proposed mass transport models for MacEtch. In Model I, Si atoms diffuse through the metal catalyst and are oxidized and etched at the metal/solution interface. In Model II, reagents and by-products of etching diffuse along the metal/Si interface. In Model III, reagents and by-products of etching diffuse through the porous metal catalyst.

One of the debated topics in MacEtch is the transport of the reagents and by-products during the etching process. These can be classified into three different proposed models, illustrated as Models I, II and III in Fig. 2. The first model (Model I) describes the transport of Si atoms through the metal before they are oxidized and etched at the metal/solution interface^20^. Models II and III are more frequently cited when describing the MacEtch process, and both involve the oxidation and etching of Si at the metal/Si interface instead. The difference lies in how the reagents and by-products are transported into and out of the metal/Si interface. Model II describes this transport via the boundary of the metal/Si interface^23^,^26^,^27^,^28^ while Model III describes the transport through the porous metal film^4^,^15^,^18^.

**Figure 3:**
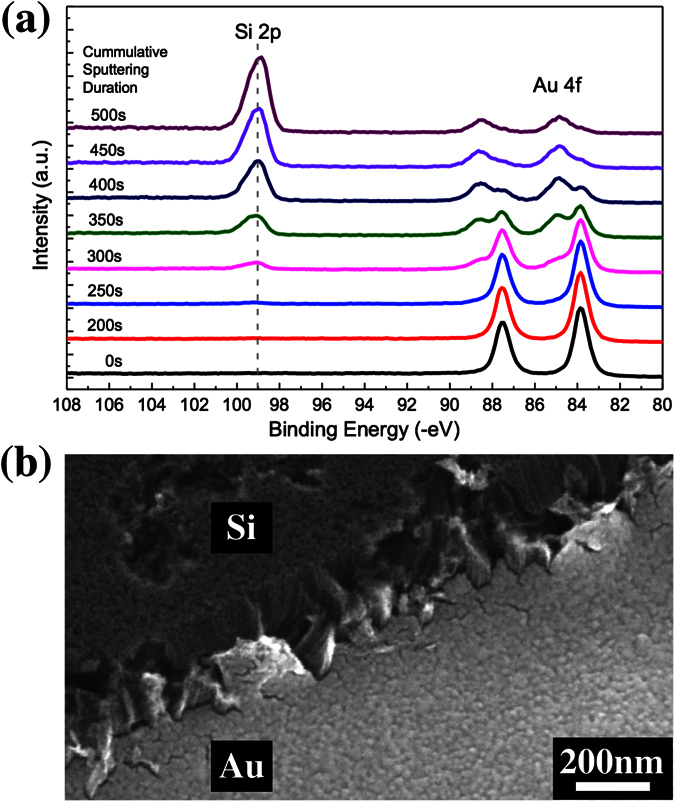
(**a**) XPS Au 4f and Si 2p core-level depth profile. The absence of any Si signal between ~98–104 eV in the bulk Au film is observed. The time indicated for each spectrum represents the cumulative sputtering duration. (**b**) SEM image at the step edge for Au dots on Si after a 20 mins MacEtch. The Au covered area is lower than that of the Si surface showing the etching of Si.

There are no direct evidences to either support or reject the first proposed model. A key support of this interpretation is the propensity for Si to mix and diffuse through a catalyst like Au^29^,^30^,^31^. The model also allows for easy etching of Si since the redox reaction now occurs at the interface with the electrolyte. In order to examine the possibility of Model I, we studied the chemical depth profiles of etched samples using XPS. We fabricated 40 nm thick Au dots on Si using shadow masking and the samples were subjected to a 20 mins MacEtch process. Figure 3a shows the typical depth profile of the Au dots after catalytic etching. In the bulk of the Au film, represented by sputtering duration of up to ~250 s, there are no observable signals in the Si 2p binding energy from ~98 to 104 eV. This means that there is no Si present, either in its elemental or compound form, in the bulk of the Au film after etching. This is despite observable etching of Si, as can be seen from the step edges of the Au dots from their SEM micrographs in Fig. 3b. The peak at ~99 eV that belongs to the Si substrate can only be seen after ~300 s of sputtering. The concomitant decrease of the Au 4f core level peak, and the binding energy of Si 2p, shows that these Si core level peaks are contribution from the substrate and not embedded Si in the Au film. It is interesting to note that at the interface, Au 4f_7/2_ has a higher binding energy of ~85.0 eV. This can be contributed by the formation of Au-Si, which supports the mixing of Au and Si at the interface. While this shows that the interface is not abrupt, more studies are needed to investigate how such mixing plays a role in the MacEtch process. In any case, the lack of embedded Si in the bulk of the Au film proves that the transport process did not follow Model I.

Between the second and the third models, there have been experimental evidences that give support to each of them. Geyer *et al*.^23^ showed that the etching rate decreases with the increasing lateral sizes of Ag stripes. This suggests that the transport of the reagents and by-products occurs through the metal/Si boundaries (Model II), since the larger lateral sizes increase the transport path length. This is also supported by Liu *et al*.^27^, who reported a higher etch rate for a 112 nm thick Au nano-cube when compared with a thinner 6.9 nm thick Au nano-prism. Both authors, however, have some differing views for the nature of such transport. Geyer *et al*.^23^ proposed that the transport occurs through a porous Si layer underneath the metal catalyst, while Liu *et al*.^27^ claimed that such porous layers are not necessary. Experimental support for Model III has also been reported when the use of a porous catalyst film, as opposed to a thick film, was shown to eliminate the preferred edge etching effect that is associated with slow reagents and by-products transport^18^. Recently, it was also shown that the pores in the metal film can be created during catalytic etching of III-V compound semiconductors, and transport of the reagents and by-products through the thin porous metal have been suggested as the dominant mechanism^32^.

**Figure 4:**
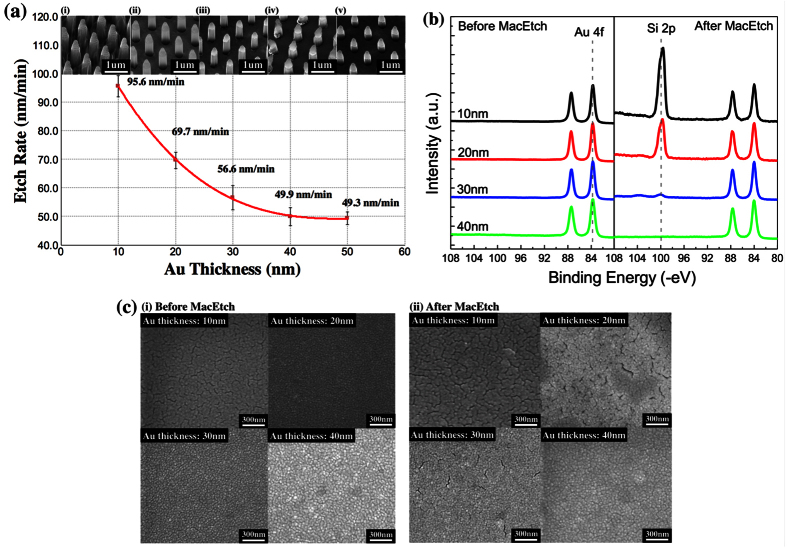
(**a**) Plot of etch rate vs. the Au catalyst thickness. The etch rate is determined from the length of the Si nanowires after a 10 mins MacEtch process, the error bar for nanowire diameter is ±15 nm. The accompanying high resolution SEM images of (i) 10 nm, (ii) 20 nm, (iii) 30 nm, (iv) 40 nm and (v) 50 nm Au thickness samples after MacEtch are shown in the insets. (**b**) Au 4f and Si 2p XPS scans before and after MacEtch. No Si 2p peaks are detected for all different Au thickness samples before the etching. After MacEtch, clear Si 2p substrate peaks are observed for the 10 nm and 20 nm Au thickness samples. A small Si 2p peak is seen for the 30 nm Au thickness sample, while no Si 2p peaks are detected for the 40 nm Au thickness sample. (**c**) SEM images of 10 nm, 20 nm, 30 nm and 40 nm thickness Au dots (i) before and (ii) after MacEtch. For the 10 nm, 20 nm and 30 nm thick Au film, the crack densities are increased after MacEtch. Only the 40 nm thick Au sample did not show any crack formation after etching.

In order to better understand this transport mechanism, we performed a detailed controlled study of the etch rate by using Au catalyst structures with differing thicknesses. The rest of the conditions such as lateral dimension, spacing, etch solution and etch duration (10 mins) were kept constant. Figure 4a shows a summary plot of the determined etch rates when only the Au catalyst thickness is varied. It can be seen that while the etch rate generally decreases when the catalyst thickness is increased, there are two distinctive segments. The decrease is more significant for Au thicknesses below 30 nm. Above which, we observe that the etch rate starts to saturate, particularly when the Au thickness is above 40 nm. The two segments show the existence of two different mechanisms at play. At smaller thicknesses, the large decrease in etch rate with increasing Au thicknesses suggests a transport mechanism of the reagents and by-products through the Au films. At thicknesses above ~30 to 40 nm, the saturation in the etch rate shows a shift towards transport via the metal/Si interface, since such mechanism will make the etch rate independent of the Au thickness. The same experimental results are similarly observed for samples with an additional photoresist lift-off step (see Supplementary Fig. S1), while the same trend is observed even for different etching duration (see Supplementary Fig. S2). In summary, we conclude that both Model II and Model III can accurately explain the transport of the reagents and by-products. We report that there is a change in the preferred mechanism from transport through the Au film for thinner catalyst, to transport through the boundaries at the Au/Si interface for thicker catalyst. This transition happens at a Au catalyst thickness of ~30 to 40 nm. We add here that observed critical thickness, if any, may be different for Ag. Both the SEM and XPS analysis (see Supplementary Fig. S3a–c) of Ag morphology before etching shows the inability of Ag to properly wet Si. In addition, Ag tends to be mobile and unstable during etching. (see Supplementary Fig. S3d,e) The instability of Ag in etching solution, together with the poor morphology makes it hard for the Ag film to remain intact and void of pores during MacEtch. These may be the reason for the unchanging etch rate dependence as observed with increasing Ag thickness by Geyer *et al*.^23^.

Supporting proof of the above-mentioned interpretation is obtained through XPS surface scans of the 10 nm, 20 nm, 30 nm and 40 nm thickness Au dots before and after the MacEtch process. Figure 4b shows the Au 4f and Si 2p scans before and after the MacEtch, respectively. As expected, no Si 2p peaks are observed before the MacEtch process for all Au thicknesses, showing good coverage of the Au dots. After the MacEtch process, the XPS scans in Fig. 4b. shows the appearance of Si 2p peaks for the 10 nm and 20 nm Au thickness dots. The Si 2p peaks consist mainly of the substrate peak at ~99 eV with small contributions from the Si-O peak at ~103 eV. The 30 nm thickness Au dots show only attenuated peaks (Si-substrate is not exposed), while no Si 2p signals are observed for the 40 nm thick film. Since the escape depth of the photoelectrons are only ~5 nm, the appearance of the Si peaks in the XPS surface scans shows that the Au dot coverage is no longer continuous after MacEtch, with presence of possible cracks or pores. The creation of cracks/pores is also ascertained by SEM images. The SEM micrographs in Fig. 4c(i) show the surface of the Au dots before etching, while Fig. 4c(ii) shows the micrographs after the MacEtch. Clear crack formation for the 10 nm, 20 nm and 30 nm thick Au film can be seen in Fig. 4c(ii) after etching. The density of cracks is also reduced as the Au thickness is increased with the 40 nm thick Au film showing no formation of cracks/pores, which is consistent with the XPS data. Images taken from an optical microscope also show, on a larger scale, increased roughening of the Au dots after etching, with the 40 nm thick Au film remaining relatively smooth (see Supplementary Fig. S4b). It should also be noted that some cracks/discontinuity are visible for the 10 nm thick Au film before etching. However, the XPS data in Fig. 4b ascertained that these are not through-pores since we cannot detect the presence of any Si core-level peaks. The optical image for the 10 nm thick Au film before etching is also smooth and continuous (see Supplementary Fig. S4a).

The roughening and formation of the cracks/pores occurs only after the MacEtch. This shows that the etching process itself introduces porosity that enabled the reagents and by-products transport. This explains the observation of the high etch rate for thin Au catalyst films. Thin Au films with thicknesses that are of 30 nm or less allow for the creation of cracks/pores that enable the transport of species through the metal (Model III). This greatly enhanced the rate of etching since this is a significantly shorter pathway when compared with the lateral sizes. Such a mechanism can explain the formation of nanowhiskers during etching with thinner catalyst films since they can be created with the porosity^23^. For Au films that are thicker, creation of a through pore/crack becomes difficult or less likely. The etch rate at such Au thicknesses therefore saturates with increasing thickness as transport of the reagents and by-products now proceeds via the boundaries of the metal/Si (Model II). We therefore conclude that both Models II and III are congruent with each other where Model III dominates at catalyst film thicknesses of ≤30 nm while Model II dominates for thicker Au catalyst films. The transport through the catalyst occurs via the formation of cracks/pores during MacEtch itself. We note here that the reported transition thickness is applicable for a continuous coverage and this will inevitably be different if the surface morphology changes. However, we believe that such microstructure evolution in affecting the etch dynamics is a general phenomenon that can be applied to different substrates as well. We investigated the trend for the etching of n-type Si (see Supplementary Fig. S5) and it agrees well with our proposed understanding. Formation of the cracks/pores were also consistent with observations in etching of GaAs and GaP for thin catalyst films^32^,^33^, while we demonstrated the lack of pores formation for the etching of GaAs using thicker Au catalyst film (see Supplementary Fig. S6). We will show subsequently that the formation of such cracks, as evidenced in this section, also represents a key supporting evidence for our proposition on the charge transport mechanism discussed in the following section.

### Charge transfer at metal/Si interface

**Figure 5:**
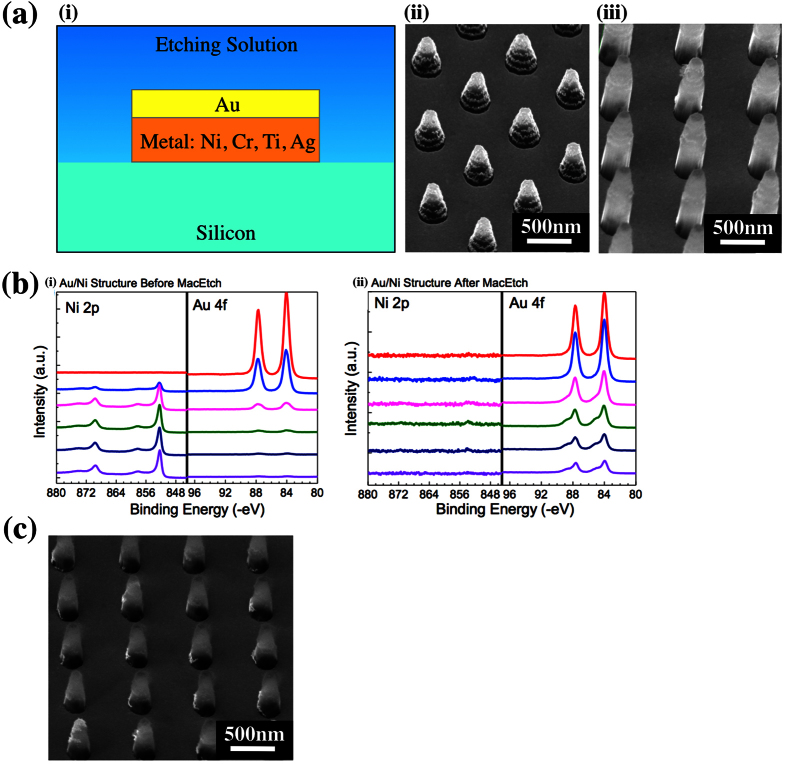
(**a**) (i) Schematic of bilayer structures used to investigate charge transfer at the metal/Si interface, (ii) Au/Ni (20 nm/10 nm) bilayer structures after 5 mins etching, and (iii) Au/Ni (20 nm/10 nm) bilayer structures after 10 mins etching. (**b**) XPS depth profiles of a Au/Ni bilayer structure (i) before and (ii) after the MacEtch process. Only a small amount of Ni is detected after etching when compared with the bilayer before etching. (**c**) Au/Ni (10 nm/20 nm) bilayer structure after 10 mins etching.

In the current understanding of the MacEtch process, holes that are catalytically generated at the surface of the metal film are then transported to the metal/Si interface, whereby Si oxidation occurs via hole injection into Si, or electron extraction from Si^26^,^34^. Since the electronic conductivity in the metal catalyst is not expected to be an issue, the transport barrier at the interface appears to be the limiting factor in controlling the oxidation of Si. Therefore, in this section, we set out to investigate the charge transfer at the metal/Si interface in the MacEtch process by designing bilayer structures as shown in Fig. 5a(i). The top layer of the catalyst is designed to remain as Au to serve two functions. The first is to provide the same reduction catalyst, thus keeping the hole generation rate consistent at the etching solution interface. The second function is to act as a protection layer for the bottom metal layer. In doing so, we can now change the type of metal in the bottom layer to investigate how etching is affected when properties, such as the contact barrier, is altered. Our group has previously shown that such a bilayer structure using Au and Cr acted effectively as an etching barrier^22^. In this work, we will extend the range of metals used as the bottom layer to include titanium (Ti), silver (Ag) and nickel (Ni). The choice of metals used is dictated by both the range of their properties, such as the metal work function or electronegativity, and the stability of the metal in the etching solution^35^.

Bilayers of Au/Cr, Au/Ag, Au/Ti and Au/Ni were used as the etching catalyst for etching duration of 5, 10 or 20 mins. We have checked the surface morphology of the single and bilayer metals to ensure that they have good coverage without any clear cracks or void. Ag layers and Au/Ag bilayers, however, have a poor starting morphology (see Supplementary Fig. S3) and Ag itself is a known catalyst for MacEtch. Thus it may not be surprising that the bilayer structure of Au/Ag produced etched Si nanowires (not shown). We will discuss more about the implication of the Au/Ag bilayer structure result in the following section, as it has a larger implication for hole transport. No etching was observed for the Au/Cr bilayer structure, as expected from our previous work (not shown)^22^. For Ti and Ni, we found that both materials inherently are etch block layers. Figure 5a(ii) shows the SEM micrographs of the Au/Ni bilayer structure after a 5 mins MacEtch where no etching is observed. This can be seen as the resultant structures are similar to the photoresist patterned structures for a sample without any MacEtch. However, after a longer MacEtch duration of 10 mins, we can start to observe the formation of the Si wires beneath these photoresist structures as shown in Fig. 5a(iii). This result is actually similar for the Au/Ti bilayer structure (see Supplementary Fig. S7) where observable etching can only be seen after a longer 10 mins duration MacEtch process. We found that subsequent etching after longer durations are actually caused by the erosion of the bottom Ni or Ti layer from the sides of the structure. The erosion of the bottom metal layer is shown by XPS depth profiles of a Au/Ni bilayer structure before and after the MacEtch process in Fig. 5b(i,ii), respectively. Figure 5b(i) shows the presence of Ni beneath the Au film before the MacEtch. However, after the etching, only a small amount of Ni can be detected as shown in Fig. 5b(ii). This shows the erosion of the bottom Ni layer during the MacEtch process. This erosion can be judged to occur from the edges of the structure based on two observations. Firstly, XPS scans showed good coverage of Au on both Ti and Ni where no elemental peak (Ti or Ni) from the bottom layer is detected from surface scans (not shown). Secondly, we fabricated micron-sized marker patterns of the Au/Ti bilayer structure using optical lithography (see Supplementary Fig. S8a.) and showed that Ti can effectively block the etching of the structure (see Supplementary Fig. S8c). The large marker pattern, as a whole, remains unetched, even though some possible etching at the edges of the structure can be detected. This is immediately obvious when compared with an identical structure (15 nm thick Au) that is etched without protection from Ti (see Supplementary Fig. S8b). Therefore, as expected, we can effectively delay the onset of Si etching by increasing the thickness of the bottom blocking metal. When the thickness of Ni is increased from 10 nm to 20 nm, we observed shorter etched Si wires after the 10 mins MacEtch as shown in Fig. 5c.

The inherent blocking capability of Ti and Ni is important as we examine the fundamental reason behind the inhibition of charge transfer at the Ti/Si, Ni/Si or Cr/Si interfaces. Table 1 shows the properties of silicon and the different metals used in this work. From Table 1, we can come to a conclusion that both the work function and electronegativity of the metal are not fundamental parameters that govern the etching process.

The work function of a metal is perhaps the most straightforward parameter to consider when it comes to the metal/Si interface. Indeed, it has been suggested that the type of transport barrier at the metal/semiconductor interface (i.e., Schottky or Ohmic) will control the charge transfer and hence the MacEtch process^34^,^36^,^37^,^38^,^39^. On perusal, this appears to be true. For hole transport, Au with a work function higher than the ionization potential of Si (5.12 eV) forms an Ohmic contact with Si, while metals like Cr, with a work function of ~4.50 eV should yield a Schottky barrier. Thus, this is consistent with the prohibition of hole injection when Cr is used as the bottom layer, thereby stopping charge transport in the MacEtch process. However, this understanding is based on the Schottky-Mott rule. In reality, Si belongs to the covalent class of semiconductors that has high electron polarizability that results in strong Fermi-level pinning based on the metal-induced gap states model^40^. The model predicts that the work function variation of 4 to 5.4 eV gives only a linear barrier height variation of ~0.85 to 0.96 eV, regardless of the Si doping. It is therefore unlikely that such small variations (~0.1 eV) in barrier height can yield drastic differences in hole injection properties at the metal/Si interface. This can be shown by doing a simple comparison of a Au/Si/Au structure versus a Au/Cr/Si/Au structure (see Supplementary Fig. S9). It can be seen that while there is a higher resistance for the Au/Cr contact, it is essentially still an Ohmic barrier and catalytic etching should not be totally inhibited. In addition, our findings in this work as summarized in Table 1, give clear evidence to the interpretation that the work function, or the transport barrier, does not govern the charge transfer process at the metal/Si interface. Specifically, Ni, with a work function similar to that of Au, is shown to be a stop etch layer, while conversely, Ag with a similar work function as Cr, is actually a good catalyst for MacEtch.

Apart from the work function, electronegativity has also been proposed to be the controlling parameter that governs this charge transfer process as it represents the relative propensity of different elements to attract electrons^26^. Our previous work with the Au/Cr bilayer structure also showed this to be possible since Cr, a good etch stop layer, has a much lower electronegativity when compared with Au^22^. Initial results with Ti also suggest this to be true since the lower electronegativity of Ti may indicate that it is a good etch stop layer. Unfortunately, Ni and Ag have similar electronegativities as shown in Table 1. This means that using electronegativity as a parameter similarly cannot explain why Ni is a etch stop layer while Ag is not. This might not be too surprising since electronegativity is a concept that is more suited to examine chemical bonding and thus has its limitations in the intuitive understanding of the MacEtch mechanism.

**Figure 6:**
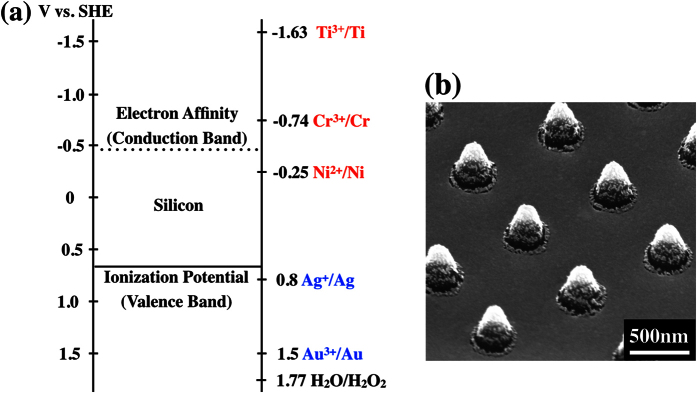
(**a**) Redox potentials of selected metals and H_2_O_2_ when compared against the ionization potential (VB: valence band) and electron affinity (CB: conduction band) of silicon. A same plot involving other elements is included in Section S6, ESI (**b**) SEM image of Au/Ni (10 nm/20 nm) bilayer structure on n-type silicon after 5 mins etching.

In this work, we find the reduction-oxidation or redox potential to be a consistent parameter in examining the charge transfer process at the metal/Si interface during MacEtch. Redox potentials have previously been shown to be consistent when considering deposition and dissolution of different metals on Si^41^,^42^. In this case, we are comparing the redox potential of the metals against that of Si. A comparison of the relative energy levels is shown in Fig. 6a, where the redox potential of the selected elements is compared against the electron affinity (conduction band) and ionization potential (valence band) of Si. It is interesting here to discuss the relevance of the electron affinity and the ionization potential of Si in the redox process. When redox potentials are compared, this is essentially a reaction driven charge transfer. In particular, since Si is oxidized, the removal of a bonding electron from the vicinity of the Si atom is necessary. The relevant energy of reference in this case is the ionization potential of Si representing the bonding states in the valence band. This should be used as the gauge for redox reactions as opposed to the mobile electron charges in the conduction band of Si, represented by the electron affinity value. This view was mentioned by Peng *et al*.^26^, as they suggested that the valence band should be the key energy potential when comparing different redox reactions. We are aware of reports that suggest that the electron injection from the conduction band of Si to be a possible route^34^. It is our view that this is not the case for the MacEtch process. An indirect evidence to suggest why this is unlikely is through the etching of intrinsic Si (resistivity of >20000 Ω-cm) with an etch rate that is comparable to that of doped Si^43^. This is a clear indication that the availability of carriers in Si is not critical in deciding the viability of the MacEtch process. In our work, we further demonstrate this by performing MacEtch using the Au/Ni bilayer structure on n-type Si substrates. The significance of this experiment can be understood from Fig. 6a. The redox potential of Ni straddles within the band gap of Si. If the etching can proceed via electron injection from the conduction band of n-type Si, we should expect etching to proceed for the Au/Ni bilayer structure. However, no etching is observed as shown in Fig. 6b. Therefore, we can conclude that the MacEtch process is dictated by the electron extraction from Si, as represented by the ionization potential (valence band).

The above discussion using redox potentials gives a more unified and consistent picture on the choice of metals that can, or cannot, be used as the contact interface with Si in the MacEtch process. The inclusion of other metals such as Pt, Fe and Cu to the redox potential diagrams are also shown (see Supplementary Fig. S10a) together with marker etching of Cu (see Supplementary Fig. S10b). It can be concluded that metals having a higher redox potential than the ionization potential of Si, generally cannot facilitate the MacEtch process. This is because the oxidation of Si is prevented by inhibiting the electron extraction from the valence band of Si. Having this understanding allows us to decide on the appropriate metal catalyst or blocking layer in the fabrication of Si nanostructures using MacEtch. Using the redox potential as a gauge is also reasonable as this indicates a reaction driven charge transfer mechanism that fits the narrative of Si oxidation for etching. With the reaction-driven mechanism, we need to examine possibilities of ion transport from the catalyst surface to the Si interface, apart from the accepted carrier transport model.

### Transport of carriers to the metal/Si interface

In the preceding section, we have concluded that charge transfer at the metal/Si interface is governed by a reaction-driven redox process. This is concluded from comparing various redox potentials of the metal catalyst that is in contact with Si. Since the oxidation process is not equivalent to an electronic carrier injection, we can question if ions transport can be used to explain the transport of charges from the catalyst surface to the Si interface.

**Figure 7:**
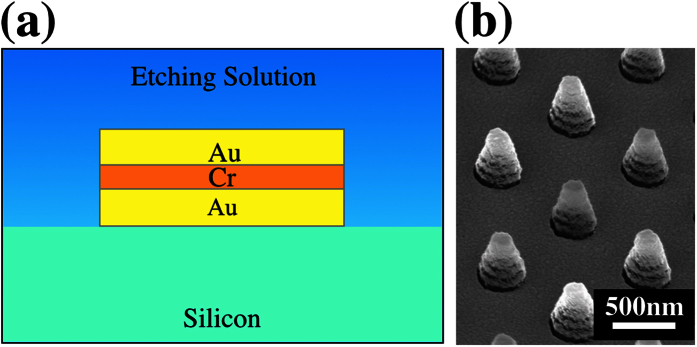
(**a**) Schematic of the trilayer structure. The top and bottom layers of the catalyst remain as Au, with Cr as the sandwiched layer. (**b**) SEM micrographs of the 10 nm Au/5 nm Cr/10 nm Au trilayer structure after a 20 mins MacEtch process.

In order to examine the above-mentioned phenomenon, we fabricated a trilayer structure of Au-Cr-Au as shown in Fig. 7a. Its surface morphology before MacEtch is also smooth without cracks or pores (see Supplementary Fig. S11a). This structure preserves both the Au-electrolyte interface and the Au-Si interface, with an added Cr layer to test the origin of the etch stop process. If Cr inhibits etching by any interface related phenomenon, we will expect the Au-Cr-Au structure to show etching of Si since a Au/Si interface is still maintained. Conversely, if the Cr in the trilayer structure can function as an etch stop layer, it must be a barrier to the transport process in general. Interestingly, as shown in Fig. 7b, such a trilayer structure does not show any catalytic etching of Si even after a 20 mins MacEtch. The same results are obtained despite using different Au thickness after lift-off of the photoresist (see Supplementary Fig. S12). Furthermore, we confirm that there is no oxidation of Cr that might have prevented the carrier transport (see Supplementary Fig. S13). The Au-Cr-Au trilayer was also demonstrated to form an Ohmic contact with Si (see Supplementary Fig. S9) and is still electrically conducting. These are significant results. The lack of etching proves that the hole transport process from the catalyst surface to the Si interface may not be accomplished by mobile hole charges since the trilayer structure did not change the electrical conductivity. Here, we propose that in the MacEtch process, the transport of the oxidized catalyst ions can be an important transport process of hole charges to the interface of Si. The roughening, pore or crack creation as reported in section (transport of reagents and by-products in etching) also agrees with this proposed understanding whereby the Au ions are mobile and diffusing. Furthermore, this further supports the finding in section (charge transfer at metal/Si interface), whereby the charge transfers at the Au/Si interface are governed by redox reactions between the Au ions and Si. The main transport process in MacEtch, at least for our experimental conditions, appears to be dominated by ion transport, and not electronic hole conduction, as will be further elaborated below.

One consideration for the ion diffusion explanation is the case of the Au/Ag bilayer structure. Since Ag is stable in the etching solution, Au ions are also prevented from reaching the interface of Si in the Au/Ag bilayer structure. In such a case, the hole charges have to be transferred to Ag as etching was observed for such a structure in the previous section (charge transfer at metal/Si interface). The charge transfer between Au and Ag can be explained by a galvanic displacement reaction between the two metals. (see Supplementary Eqs 6 to 8 in section S14) When Au ions diffuses to the Au/Ag interface, Ag atoms are oxidized in the displacement reaction in forming Ag^+^ ions and the MacEtch process can still proceed at the Ag^+^/Si interface since the redox potential of Ag allows for electron extraction from Si. This interpretation provides a detailed explanation for how some metals, like Cr, is an effective etch stop layer. When used as a bottom layer as shown in Fig. 5a(i), the displacement reaction can oxidize metals such as Ti, Ni and Cr. However, as all these metals have higher redox potentials when compared to the ionization potential of Si, charge transfer at the metal/Si interface is prevented. When used as the sandwiched layer in the trilayer structure as shown in Fig. 7a, the oxidized Cr^3+^ layer cannot reversibly oxidize the bottom Au layer, based on the anodic index in galvanic displacement reactions^44^. Therefore etching is prevented by inhibiting the transport of positive ions to the metal/Si.

### Summary of new route for the MacEtch Process

In summary, we can propose that the MacEtch process is being governed primarily by two redox and one etching reactions as follows:


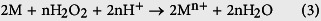



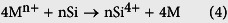



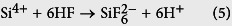


**Figure 8:**
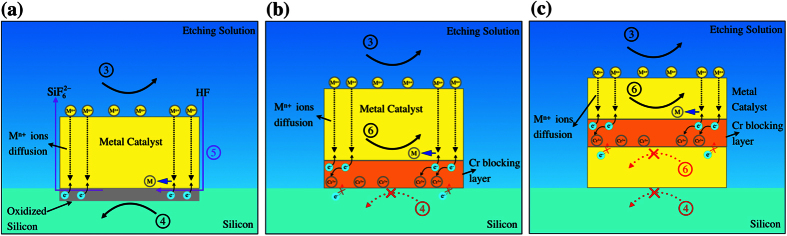
Schematics for the reactions of (**a**) single layer, (**b**) bilayer and (**c**) trilayer structures. The reaction 3 is the oxidation of metal (M) catalyst (Eq. 3), the reaction 4 is oxidation of silicon (Eq. 4), the reaction 5 is etching of oxidized silicon (Eq. 5), while the reaction 6 is the galvanic displacement reaction between metal catalyst ions and the blocking layer (see Supplementary Eq. 6 in section S14).

Figure 8a shows the schematic illustrating the above reactions. On the surface of the catalyst, M^n+^ ions are formed from the reduction of H_2_O_2_ as shown in Eq. 3. While stability in the etching solution has to be considered, viability of this redox reaction is usually favourable for many different metals as can be judged from the redox potential of H_2_O_2_ as shown in Fig. 6a. The accumulation of the ions allows for the diffusion to the Si interface whereby the second redox reaction (Equation 4) of Si oxidation occurs. To enable Eq. 4 to proceed, the selected metal at the metal/Si interface need to possess a higher redox potential versus the ionization potential of Si. Otherwise, as shown in Fig. 8b, Si oxidation cannot proceed through the prevention of charge transfer in the oxidation of Si. The inhibition of MacEtch can also be accomplished using a trilayer structure as shown in Fig. 8c. In this case, propagation of the ions is prohibited through disabling the galvanic displacement reaction between the sandwiched and bottom layers. Subsequently, the oxidized Si can now be etched (Equation 5), whereby the transport of the reagents (HF) and by-products (SiF_6_^2−^ and H^+^) will preferably occur through porosity or cracks (generated during the etching) when the Au metal catalyst is ≲30 nm thick, or via the metal/Si boundaries when the metal catalyst is thicker.

This summarized interpretation of the MacEtch process now relies only on redox reactions. Since it is possible for the process to be void of hole/electron carrier transport in the catalyst, the new understanding helps to reconcile the different mechanisms proposed in HF/AgNO_3_ etching and MacEtch. In HF/AgNO_3_ etching, also a catalytic etching process, it is the Ag^+^ ions that drive the oxidation of Si on the substrate surface. Recently, Geyer *et al*.^45^ has attempted to reconcile the two processes by suggesting that Ag films are partially dissolved as Ag^+^ ions in a solution. It is the Ag^+^ ions in the solution that mediates the hole carrier transport. Unfortunately, our attempt to quantify the Au ions in the solution by inductively coupled plasma mass spectrometry was not successful due to the low concentration level of Au ions and the dilution needed for the technique. Nonetheless, our work suggests that such mediation need not be evoked as we show the possibility of the direct ion transport to the Si interface. The two etching processes are thus fundamentally similar. The only difference may be the diffusion process of the ions whereby MacEtch will rely on solid-state diffusion instead. This new understanding will also explain discrepancies between the work from Mikhael *et al*.^46^, who observed the presence of Au particles on the sidewalls of fabricated SiNWs, and that from Chiappini *et al*.^47^, who observed otherwise after etching by nanoparticle catalyst. In the redox driven charge transfer as described in this work, the presence of Au particles for the former is more likely since a redistribution is necessary for the re-oxidation of the reduced Au ions in thin films. On the contrary, in nanoparticles or metal ion etching, the reduced ions will have greater access to the electrolyte and thus can be easily re-oxidized. This can also be inferred from the SEM images of the Au/Cr/Au structures before and after etching (see Supplementary Fig. S11). The roughening or etching of the off-catalyst area (bare Si) shows the possibility of Au ions deposition and re-oxidation. The ion diffusion, as a transport mechanism, also explains the formation of cracks or pores after catalytic etching that is similarly observed for III-V semiconductors^32^. This is because the nature of ion diffusion, as opposed to pure carrier conduction, accounts for the mobility of the atoms during the etching process. We believe that the crack formation is a result of the dynamic redistribution of Au in the cyclic formation of Au^3+^ ions and Au, that eventually leads to the formation of cracks/pores. However, we do not rule out other possible contributing factors in the formation of the cracks or pores. Mechanical stresses from unequal etch rates may possibly cause some crack formation for micrometer size structures^4^,^15^, while other causes including the pressure created from hydrogen generation and the transport of reagents/by-products can all add to the dynamic redistribution process in the eventual formation of pores.

The new understanding in the ion transport also shows the minimal role played by the mobile carriers. This can also shed light in some observed phenomena in MacEtch. For example, in the etching process, pit formation is often observed in off-metal areas, with the pit density increasing with higher oxidant concentration. There have been debates about the origin of such pits and they can either be described by a ion re-deposition process^17^,^45^,^48^,^49^ or faster re-distribution of mobile hole carriers^20^,^50^,^51^. Our work gives stronger support to the ion re-deposition process since we are essentially looking at redox processes. The metal ions etching around the metal catalyst may also be the source of porosity creation in the etched Si, especially when there are unequal and varying etch rates in different microscopic regions^51^,^52^. Such porosity generation was also observed in our work along the walls and beneath metal catalyst (see Supplementary Fig. S15) and will be an interesting future work to test if they can be controlled or manipulated. We must add here that our work does not strictly rule out re-distribution of the holes after Si oxidation, especially if the oxidized Si is not immediately etched. However, in a recent work by Kim and Oh, the demonstrated preferred inverse etching microns away from the micron-sized nanomesh catalyst provided added support to the ion re-deposition process^33^. This is because it may not be easy to explain the preferential diffusion of mobile holes over a significantly larger distance outside of the nanomesh. Our work can also explain the observations of the etch rate when MacEtch is performed under an applied bias. When a negative bias is applied, this should significantly increase the hole injection rate, and thus the etch rate, at the metal/Si interface. However, under a negative applied bias, reported works have shown either just a small increase^53^, or on the contrary, reducing etch rates^50^. While the effects on ion movements through such applied bias is yet unclear, our work shows clearly why there is no substantial increase in etching since the charge transfer is a redox driven reaction. The redox driven reaction is also consistent with the interpretation of the role of van der Waals forces between the catalyst and semiconductor that is largely accepted^54^,^55^,^56^. The ion transport and redox reaction model is more congruent with such interface forces as efficient charge injection will require a solid state contact instead. The affirmation of the van der Waals forces will help greatly in the understanding and design of isolate catalyst etching for trenching applications, an area of work we will share in our future communication. The validity of van der Waals forces also gives added support for the validity of mass transport across the interfaces that we have found to be dominant for larger catalyst thicknesses. The understanding of the reagents and by-products transport will have implications in creating deep trenching for large structures. Previously, to prevent the problem associated with the diffusion path length in large structures, nanoporous Au can be used for the etching^18^. Our work suggests that an appropriate metal catalyst thickness is sufficient since porosity can be subsequently generated. We have also indirectly demonstrated this through our control marker experiments (see Supplementary Fig. S8b). The utilization of such knowledge will simplify the process steps required in the bulk micromachining of Si^15^,^18^. For the carrier transport and redox mechanism that we have proposed in this work, they will have important consequences in the basic understanding and unification of MacEtch processes in general. This can possibly help further studies in the control of porosity^57^, development of self-assembled lithography with different metals^58^, design of new process in etching and new etching recipes for multiple compound semiconductors^32^,^39^,^59^.

## Conclusions

This work provides clear evidences in the transport processes of metal-assisted chemical etching (MacEtch). It is shown that transport of reagents and by-products are accomplished through pores/cracks created during etching when the catalyst film is ≲30 nm thick. When the catalyst film is thicker, the transport is accomplished via the boundaries of the metal/Si interface. The creation of such pores/cracks is a result of solid-state ion diffusion in the dynamic redistribution of the ions. In our MacEtch setup, we provide evidences to show that the ion transport process is dominant over carrier transport from the catalyst surface to the Si interface. Once the transport of the metal ions to the metal/Si interface is successful, the redox potential of the ions, relative to the ionization potential of Si, will decide if the charge transfer and Si oxidation can proceed. The new understanding reconciles solution ions and thin film etching, where it also provides explanations into phenomena such as metal ions re-deposition, effects of applied bias and pits formation. The fundamental understanding of the parameters that governs the MacEtch process will provide better options in choosing suitable metal or etch block layers in the design of such processes for a variety of applications.

## Methods

Si (100) substrates used for MacEtch are boron doped p-type Si, with resistivity in the range of 0.1 to 1 Ω-cm, and phosphorous doped n-type Si, with resistivity in the range of 1 to 10 Ω-cm, that were cut into square pieces, each with area of 1.5 × 1.5 cm^2^. The samples were pre-cleaned by sonication in acetone and isopropyl alcohol (IPA), both for a duration of 15 mins each. Following this, the cleaned samples were rinsed in DI water and blown dry by a nitrogen gun. The removal of the native oxide on the Si substrate surface is not necessary since the catalytic etching is not affected by the presence of the thin oxide layer^22^. Subsequently, periodic nano-dot arrays of photoresist (S1805) were patterned by laser interference lithography. The nano-dots have a height of ~400 nm, diameter of ~350 nm and spacing of ~500 nm. A schematic illustration of the fabrication steps is shown in Fig. 1. Different metals were evaporated onto the Si substrate pieces using a Denton electron beam evaporator operating under a chamber pressure of 1 × 10^−6^ Torr, with deposition rate at 0.5 Å/s. The nominal thickness is measured *in-situ* by a quartz crystal microbalance that was calibrated for thicker Au films. For all multiple layered metal structures, the deposition was performed sequentially in the electron beam evaporator without breaking vacuum. Since the metal film thickness used in this work is significantly thinner than the height of the photoresist nano-dots, a subsequent photoresist lift-off process is not necessary. The catalytic etching was conducted under a yellow light environment in a solution consisting of 4.6 M HF, 0.15 M H_2_O_2_ and deionized water for durations of 5 to 20 mins, depending on the experimental requirement. The etched samples were rinsed in DI water before drying on a hot plate at 90 °C. Microscopy analysis was performed using a high resolution Scanning Electron Microscope (Nova NanoSEM 230). The average height of nano-pillars in our work is calculated from the 50° tilt view SEM micrographs from sample sets of 20 nano-pillars. The actual average height of the nano-pillars is therefore taken as the average height/Sin (50°). Cross-sectional SEM was performed for the structures etched with 20 nm and 40 nm Au thickness to ascertain the accuracy in using this approach (see Supplementary Fig. S16). The Chemical analysis and depth profiling were achieved using a VG ESCALAB 220i-XL X-ray photoelectron spectroscopy (XPS) system equipped with a monochromatic Al *Kα* (1486.6 eV) source, magnetic immersion lens and a depth profiling Ar^+^ gun. MacEtch samples that underwent the depth profiling analysis were structures of circular dots with diameters ranging from 80 to 1000 μm. These samples were fabricated with a shadow mask and they were used to ensure that the etched area is bigger than the X-ray spot size of ~500 μm in diameter.

## Additional Information

**How to cite this article**: Kong, L. *et al*. Evidences for redox reaction driven charge transfer and mass transport in metal-assisted chemical etching of silicon. *Sci. Rep.* **6**, 36582; doi: 10.1038/srep36582 (2016).

**Publisher’s note:** Springer Nature remains neutral with regard to jurisdictional claims in published maps and institutional affiliations.

## Supplementary Material

Supplementary Information

## Figures and Tables

**Table 1:** Work function, electronegativity and reduction potential referenced to standard hydrogen electrode (SHE), of silicon and selected metals.

Material	Work Function (eV)	Electronegativity	Reduction Potential (V/SHE)	Etching
Au	5.10	2.54	Au^3+^ + 3e^−^ → Au (1.50 V)	Yes
Ag	4.26	1.93	Ag^+^ + e^−^ → Ag (0.80V)	Yes
Cr	4.50	1.66	Cr^3+^ + 3e^−^ → Cr (−0.74 V)	No
Ti	4.33	1.54	Ti^2+^ + 2e^−^ → Ti (−1.63 V)	No
Ni	5.15	1.91	Ni^2+^ + 2e^−^ → Ni (−0.25 V)	No
Si	4.75^a^	1.90	Electron affinity (−0.46 V)Ionization potential (0.67 V)	N.A.

Their ability to facilitate MacEtch is shown in the last column.

^a^The work function of silicon is calculated on the basis of p-type silicon with resistivity in the range of 0.1 to 1 Ω-cm.
