# Supplementary material for: Evidences for redox reaction driven charge transfer and mass transport in metal-assisted chemical etching of silicon
Source: Sci Rep. 2016 Nov 8;6:36582. doi: 10.1038/srep36582 (PMC5100464; doi:10.1038/srep36582)
Supplement: Supplementary Information [file srep36582-s1.pdf]

Supporting Information

Evidences for redox reaction driven charge transfer and mass transport in metal-assisted chemical etching of silicon

Lingyu Kong,[1, 2, 3] Binayak Dasgupta,[1, 2] Yi Ren,[2] Parsian K. Mohseni,[4] Minghui Hong,[3] Xiuling Li,[4] Wai Kin Chim[3*] & Sing Yang Chiam[2*]

[1]*NUS Graduate School for Integrative Sciences and Engineering, National University of Singapore, 28 Medical Drive, Singapore 117456.*

[2]*Institute of Materials Research and Engineering, A*STAR (Agency for Science, Technology and Research), 3 Research Link, Singapore 117602. E-mail: chiamsy@imre.a-star.edu.sg*

[3]*Department of Electrical and Computer Engineering, National University of Singapore, 4 Engineering Drive 3, Singapore 117583. E-mail: elecwk@nus.edu.sg*

[4]*Department of Electrical and Computer Engineering, Micro and Nanotechnology Laboratory, University of Illinois at Urbana-Champaign, Urbana, Illinois 61801, United States.*

Correspondence and requests for materials should be addressed to S.Y.C (email: chiamsy@imre.a-star.edu.sg) or W.K.C (email: elecwk@nus.edu.sg)

S1. Au thickness dependent etch rate study after photoresist nano-dots lift-off

The lift-off step is omitted in this work to simplify the fabrication process. However, we still examine the Au thickness dependent etch rate with the lift-off step for comparison. All samples with different thickness of Au are subjected to 20 mins catalytic etching in 4.6 M HF, 0.15M H_2O_2 and deionized water. Figure S1 shows the SEM images of etched silicon nanowires (SiNWs) of samples with different Au thickness. It can be observed that the SiNWs length generally becomes shorter (etch rate generally decreases) when the catalyst thickness is increased. The aforementioned trend agrees with the etching results of samples without photoresist lift-off.

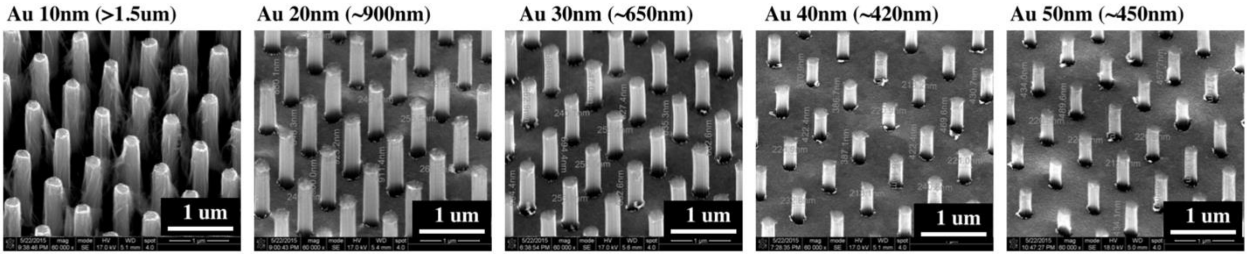

Figure S1 High resolution SEM images of (a) 10 nm, (b) 20 nm, (c) 30 nm, (d) 40 nm and (e) 50 nm thickness Au samples with photoresist nano-dots lift-off step, followed by 20 mins MacEtch. The error in estimating the nanowire diameter is ±15 nm.

S2. Etch rate as function of Au thickness for different etching duration

The etch rate as function of Au thickness at different etching duration are studied. Fig. S2 demonstrates a repeatable and consistent trend of decreasing etch rate below some critical thickness before saturation. The slower etch rate for a longer duration shows some form of saturation in the etching process with increasing etching duration. The clear trend observed with the different Au thickness across the etch durations gives us good confidence of the proposed model in this work. It worth to note here that the thicker Au films yielded some delamination after the 50 min etching and thus the data points were not shown.

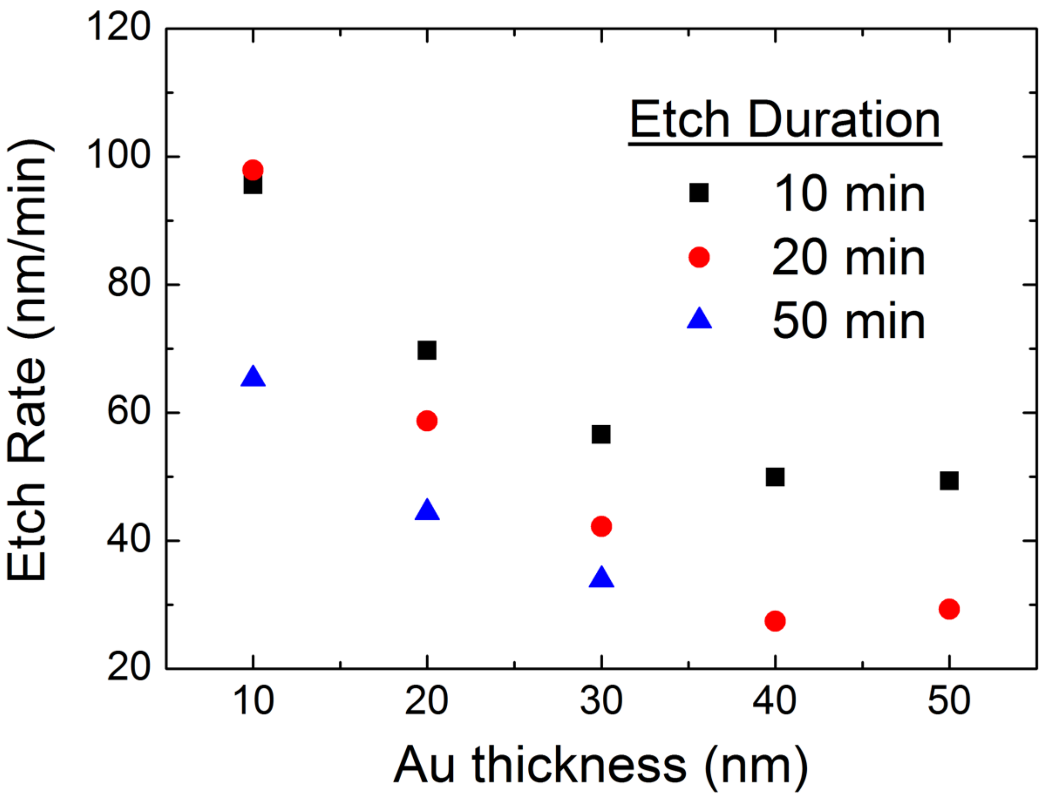

Figure S2 Plot of etch rate as function of the Au catalyst thickness for three separate etching duration. The thicker Au films showed delamination for the 50min etch duration and thus were not shown in the plot.

S3. Analysis of Ag coated Si sample before and after MacEtch

Ag has poor morphology on Si and is not stable during MacEtch. The poor wetting ability can be observed from XPS studies of the 10nm and 15nm Ag deposited on Si shown in Figs. S3a and S3b below. The presence of the Si-Si peak from the Si substrate shows the exposed Si substrate even before etching. This is made clear by the resolvable spin-orbit splitting of the $Si2p_{3/2}$ and $Si2p_{1/2}$ that would not be possible if it is a buried interface. The SEM image for a 20 nm Ag coated Si is also shown in Fig. S3c whereby the agglomeration of the Ag metal clearly expose the underlying Si substrate. The high mobility or dissolution of Ag during etching can be seen from the SEM images of patterned 10 nm Ag disc before and after MacEtch, as shown in Figs. S3d and S3e, respectively. The initial Ag disc patterns can no longer be seen while some network of Ag can be observed.

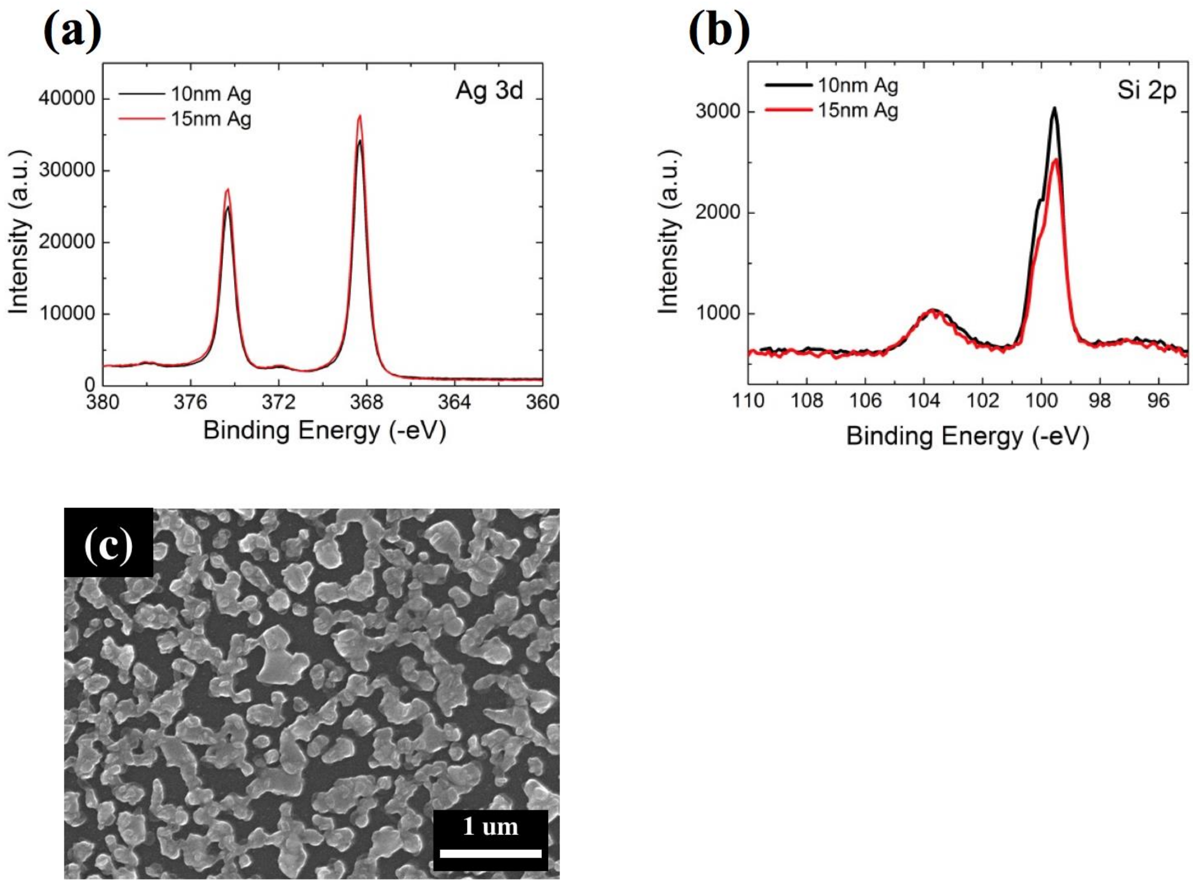

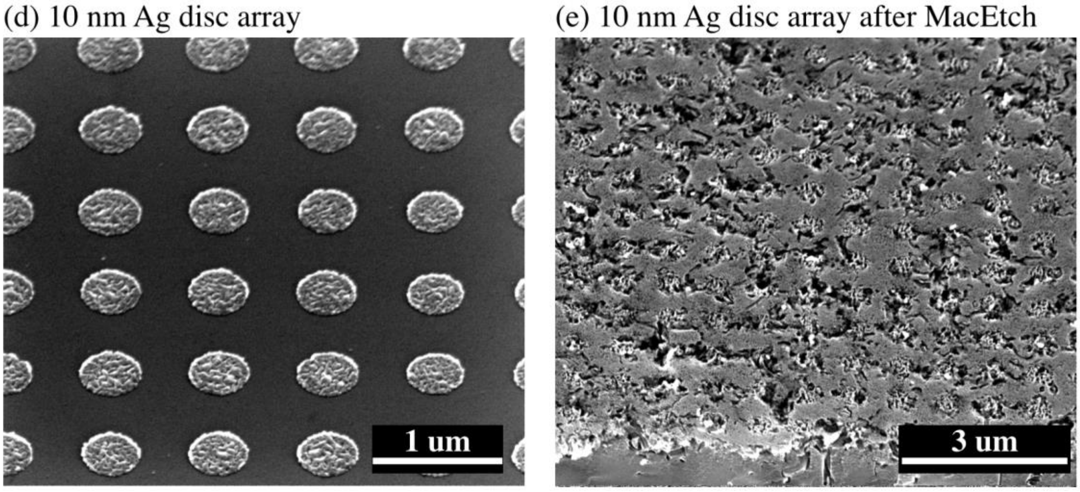

Figure S3 XPS spectra of (a) Ag 3d and (b) Si 2p for a 10 and 15 nm coated Si, (c) SEM micrograph of 20 nm Ag coated Si substrate, and 10 nm Ag disc array (d) before and (e) after MacEtch.

S4. Optical microscopy images of Au dots showing the surface roughness before and after MacEtch

Images are taken from an optical microscope before and after a 20 mins MacEtch process, for different indicated Au thicknesses. Fig. S4a shows smooth Au films before etching for all Au thicknesses. Fig. S4b shows roughening of the films for the 10 nm, 20 nm and 30 nm thick Au film. The highest roughness is observed for the 10 nm thick Au film. The 40 nm thick Au film shows a relatively smooth surface even after MacEtch.

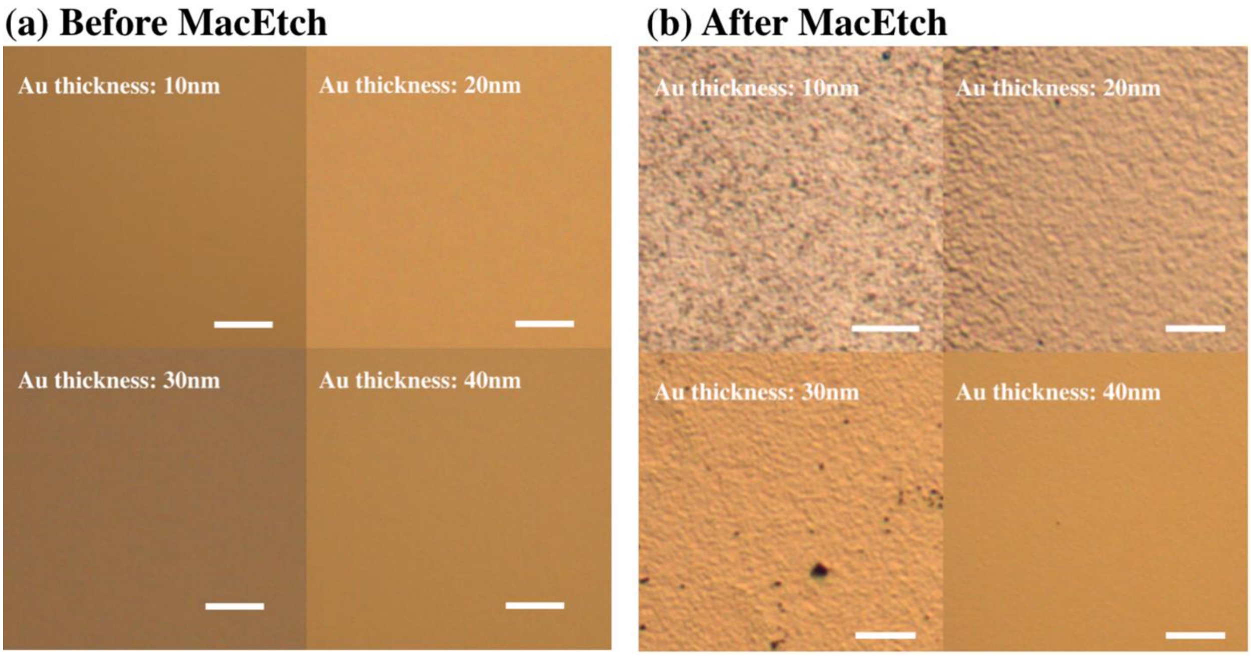

Figure S4 Optical microscopy images of 10 nm, 20 nm, 30 nm and 40 nm thickness Au dots (a) before MacEtch, and (b) after MacEtch. The inserted scale bars represent 50 μm.

S5. MacEtch results of n-type Si by nano-sphere lithography

MacEtch results of n-type Si using 20 nm Au, 40 nm Au and the Au/Cr bilayer by using nano-sphere lithography[1] are shown in Fig. S5, respectively. The results show that the 20 nm Au catalyst yielded longer pillars (~630 nm) while the 40 nm Au catalyst yielded shorter (~410 nm) pillars as expected. The results show that our proposed interpretation of the mass transport phenomenon of is not affected by the type of doping for Si as the same trend is observed in the etch rate variations. The Au/Cr etching shows again the validity of our redox model for the etching of n-type Si. Overall, the results also show that the type of lithography do not affect the proposed model either in mass transport or charge transfer.

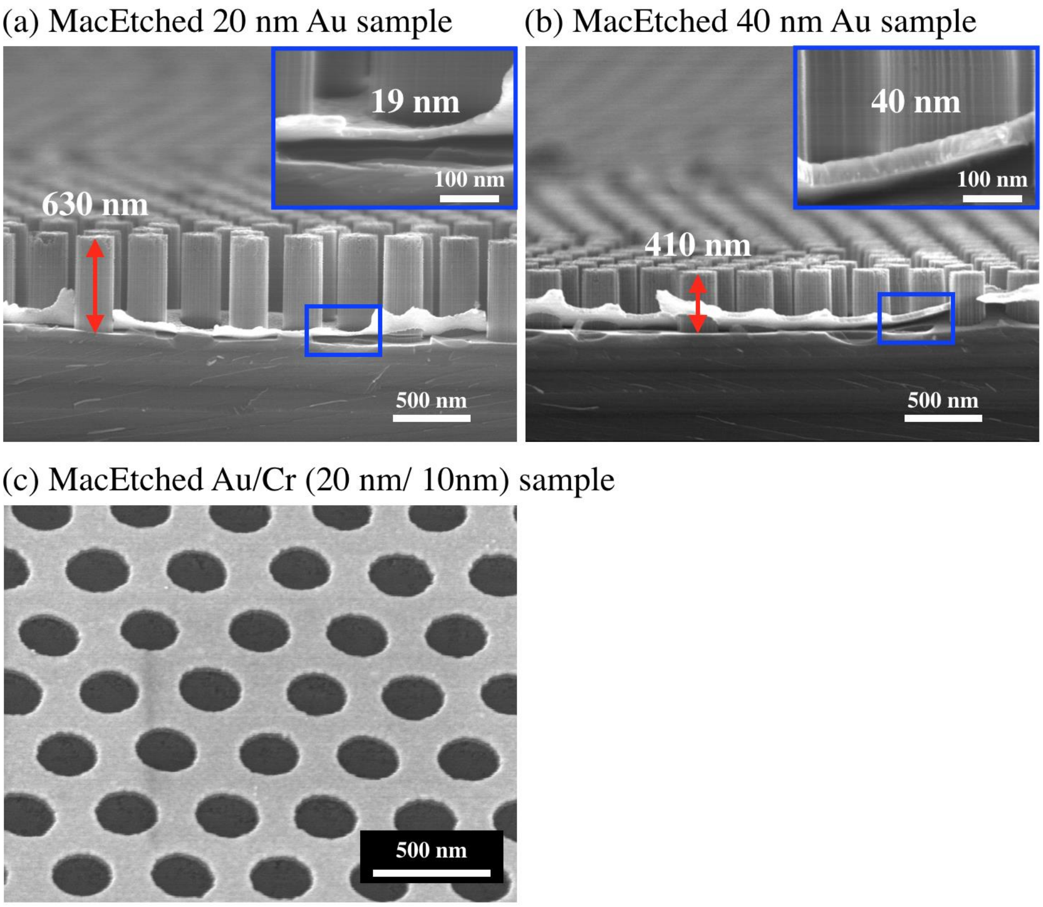

Figure S5 SEM micrographs of (a) 20 nm Au, (b) 40 nm Au and (c) Au/Cr – 20 nm /10 nm bilayer sample after 10 mins MacEtch in 4.6 M HF and 0.15 M H_2O_2.

S6. MacEtch results of compound semiconductor - GaAs

MacEtch of GaAs (n+, Si-doped) sample, using 30 nm of Au mesh was performed. The MacEtch solution consists of 14.2 M HF and 5.3 mM $KMnO_4$. As shown in the Fig. S6, no obvious pin-holes or cracks are observed on the thicker Au film, consistent with the model proposed in this work. In addition, we can observe roughening of the Au catalyst that can be attributed to the dynamic redistribution of the Au ions during etching.

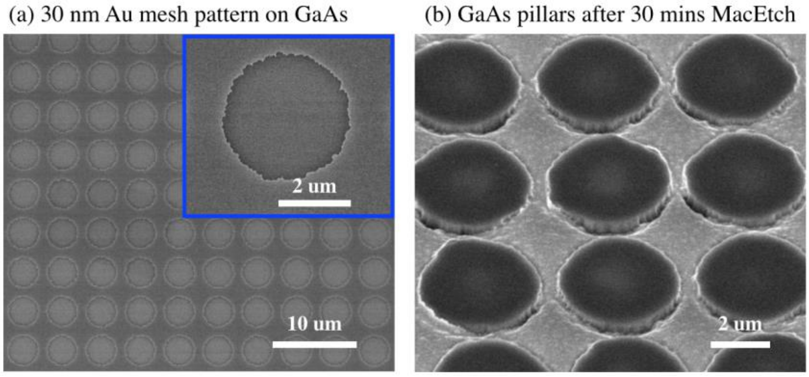

Figure S6 SEM micrograph of (a) optical lithography patterned Au mesh on GaAs and (b) GaAs pillars after 30 mins MacEtch in solutions consists of 14.2 M HF and 5.3 mM $KMnO_4$.

S7. MacEtch results of Au/Ti bilayer structure

Fig. S7a shows the SEM micrographs of 20 nm Au/10 nm Ti bilayer structure after a 5 mins MacEtch. Little or no etching is observed. However, after a longer MacEtch duration of 10 mins, the formation of Si wires can be seen as shown in Fig. S7b. These results are qualitatively similar to those obtained from Au/Ni bilayer structures of the same thicknesses.

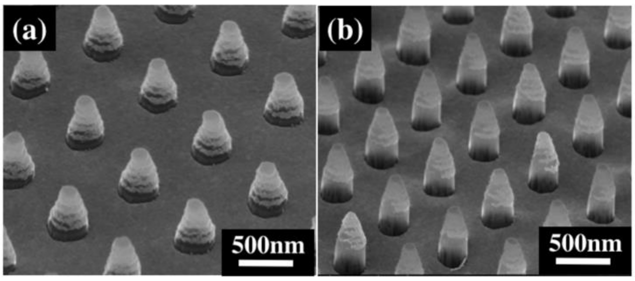

Figure S7 20 nm Au/10 nm Ti bilayer structures after MacEtch process for (a) 5 mins, and (b) 10 mins.

S8. Verification of the blocking effect of Au/Ti bilayer structure

Micron-sized marker patterns that were fabricated by standard optical lithography were used for the test of MacEtch for large patterns. These markers were protected by 10 nm Cr and 30 nm Au that are known to prevent the MacEtch process. The rest of the area is covered with the test catalyst bilayers of 10 nm Au (top layer)/5 nm Ti (bottom layer). An example of such structures before etching is shown in Fig. S8a.

A control experiment with the same marker structure, but using instead 15 nm thick Au catalyst as the etching metal, is also fabricated. Fig. S8b shows the SEM micrograph of the control experiment after a 10 mins MacEtch process. Anisotropic etching of Si was observed and the marker remains unetched.

Fig. S8c shows the result when the 10 nm Au/5 nm Ti bilayer structure is used as the etching catalyst. There was no observable etching when compared to the Cr/Au markers after a 10 mins MacEtch process. If any, etching near the edge of the markers can be observed. The lack of observable etching after an increase in the length scale of the structures showed that erosion of the bottom metal layer occurs from the edge of the structures. The etching of such large structures is thereby prevented. We also note that the etching of such large structures is also possible when using a Au thickness that allows for the creation of pores/cracks. This is so that diffusion of the reagents/by-products is not an issue for the MacEtch process.

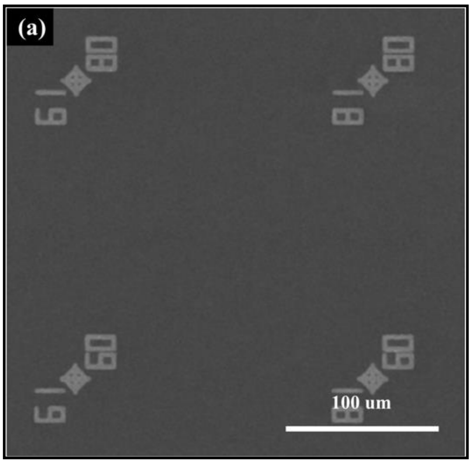

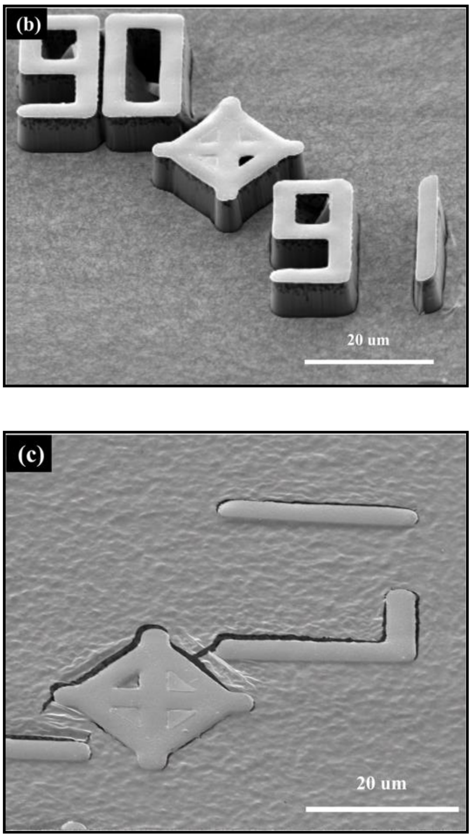

Figure S8 (a) SEM image of markers made from thermally evaporated Cr (10 nm thickness) and Au (30 nm thickness) patterned using standard optical lithography. (b) SEM image demonstrating the anisotropic etching of Si with 15 nm thickness of Au as the control catalyst. (c) SEM image demonstrating the inherent etch blocking capability of Au/Ti bilayer structure. Although no etching is observed as a whole, there can be some etching near the edges of the markers and roughening of the metal layers were observed.

S9. Electrical characteristics of contact barriers

The electrical contact properties with different metals can be tested by fabricating different top contact probes. Top contact probes of 500 μm are fabricated by shadow masking while a blanket Au (50 nm) is deposited on the back side of the wafer. Au (30 nm), Au(10 nm)/Cr (10 nm)/Au (10 nm) and Au (20 nm)/Cr(10 nm) are used as the top metal contact. Electrical measurement was performed using the Cascade Microtech probe station with an Agilent 4156C semiconductor parameter analyzer. As shown in Fig. S9, good Ohmic properties is observed for the Au-Si-Au showing the successful fabrication procedure. The Au/Cr top contact yielded higher resistance in the positive voltages due to slight depletion effects. However, it remains an Ohmic contact with the plot clearly passing through the origin. The Au/Cr/Au top contact yielded identical current-voltage curve showing the absence of any additional electrical transport barriers and that carrier transport in this structure is not impeded.

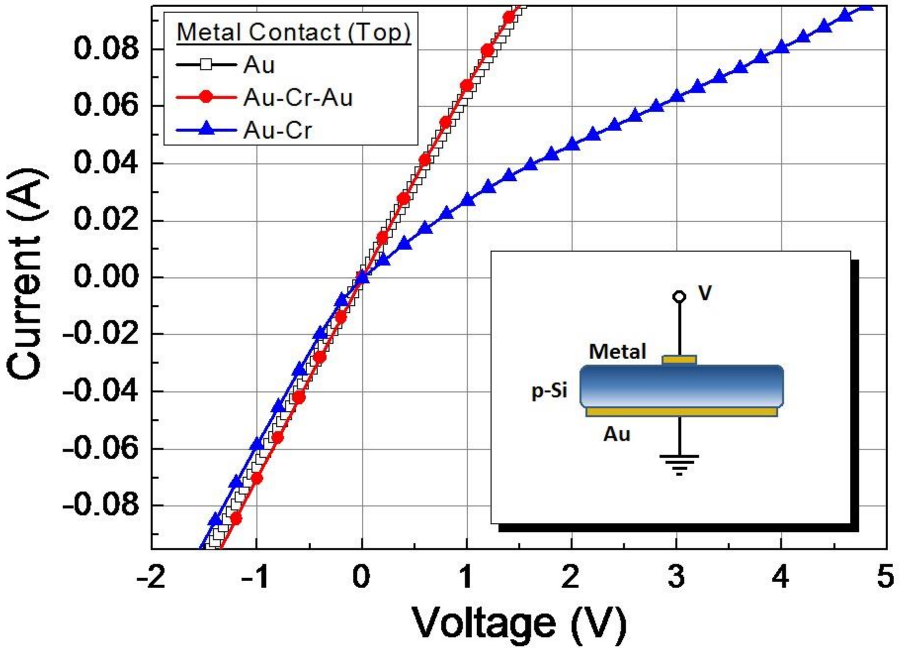

Figure S9 Current-voltage plot for different top metal contacts on p-Si with a Au blanket back contact. Inset shows the schematic of the measurement structure.

S10. Etching with Pt, Cu and Fe

MacEtch using copper (Cu), iron (Fe) and platinum (Pt) can similarly be explained with redox potential and this is shown in Fig. S10a. It is well reported in the literature that Pt is one of the commonly used catalysts for MacEtch of silicon nanostructures and thus this experiment is not repeated here.[2-4] Interestingly, Fe is also used for etching of Si through the use of $Fe(NO_3)_3$ solution.[3,5] In this case, the etching was shown to be promoted and enhanced by Ag coated Si. The Ag acts as a concentration point for the redox reaction to take place between the Fe and Si. For Cu catalyst, we examine possible blocking effect of Cu on a marker sample. The metal films deposited on the marker samples in this case consist of 15 nm Au (top layer) and 10 nm Cu (bottom layer). No clear etching of Si was observed (Fig. S10b) indicating that Cu can function as a blocking material. This agrees generally with available literature where only slight etching occurs with Cu catalyst, yielding shallow pits instead.[3,5-7] Similar to the $Fe(NO_3)_3$/Ag/Si system, Cu can act as a concentration point for the redox reaction between H_2O_2 and Si. In addition, for both etching systems, proximity of the reduction potential of a redox pair may cause an overlap of the potentials if these can be described by a Gaussian distribution.[8,9] This can similarly account for slow etching of Si using Cu as a catalyst, and the use of $Fe(NO_3)_3$ as an oxidizer for Ag, in comparing redox reactions.

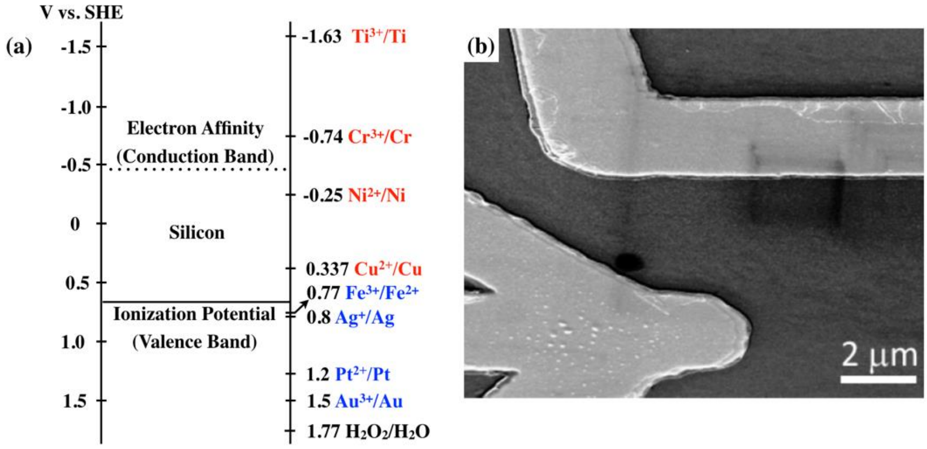

Figure S10 (a) Redox potentials of selected metals, including Cu, Fe and Pt versus silicon. (b) SEM image showing insignificant etching of Si using Au/Cu bi-layers marker.

S11. SEM comparison of tri-layer structure (Au/Cr/Au) before and after etching

The comparison Au-Cr-Au sample before and after MacEtch are shown in Fig. S11. This is a sample that do not show any etching and thus we are focusing on the holes that reveal the exposed Si. Fig. S11a below shows clearly that the exposed Si looks smooth before MacEtch. After a 20 mins dip of the sample into the etchant solution, Fig. 11b shows that the exposed Si is rougher and looks to be slightly etched. The observed phenomenon can be attributed to out-diffused Au ions and subsequent redox reaction. But this phenomenon is probably self-limiting as the oxidation and dissolution of metal ions can reach an equilibrium.

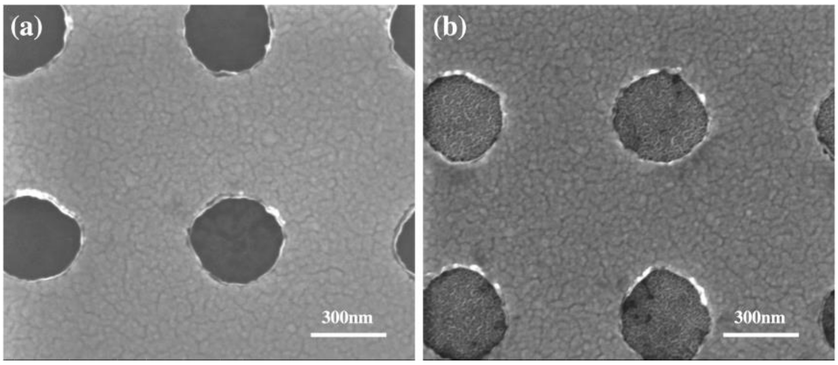

Figure S11 High-resolution SEM micrographs of Au-Cr-Au (10 nm-5 nm-10 nm) after a lift-off process, (a) before and (b) after 20 mins MacEtch.

S12. Verification of the blocking effect of tri-layer structure with different bottom Au thickness

The tri-layer structure MacEtch experiment is repeated with thicker bottom Au thickness after photoresist lift-off. This is to ensure that there are no Cr-Si contact and also that the photoresist does not hinder the mass transport process for such thicknesses. The sample thicknesses of the tri-layer structures are as follows:

1. Si/**Au**-Cr-Au (**10 nm**-5 nm-10 nm)
2. Si/**Au**-Cr-Au (**15 nm**-5 nm-10 nm)
3. Si/**Au**-Cr-Au (**20 nm**-5 nm-10 nm)

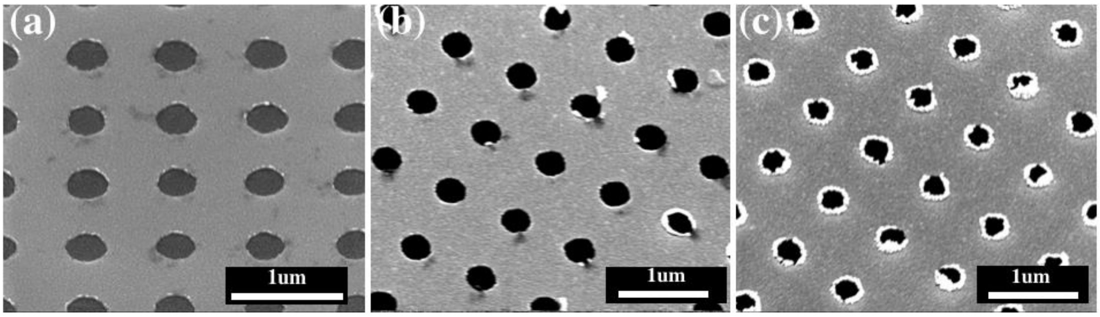

Figure S12 SEM micrographs of tri-layer structure with different bottom Au thickness after lift-off process and followed by 20 mins MacEtch. (a) Au-Cr-Au (10 nm-5 nm-10 nm), (b) Au-Cr-Au (15 nm-5 nm-10 nm) and (c) Au-Cr-Au (20 nm-5 nm-10 nm).

As shown in Fig. S12, no etching is observed for all thickness variation after 20 mins MacEtch. This further gives support to the proposed model on ion transport.

S13. XPS depth profile analysis of tri-layer structure before and after etching

The possible oxidation of Cr is studied using XPS depth profile of 20 mins etched and non-etched tri-layer (Si/Au/Cr/Au – 15/5/10 nm) samples. The depth profile analysis of the etched sample in Fig. S13a shows the Cr $2p_{3/2}$ peak appears after 30 s of sputtering and clear metallic Cr $2p_{3/2}$ peak at 574.3 eV can be observed after 60 s. The Cr peak finally disappears after sputtering for 150 s. This is similar to the unetched sample (Fig. S13b), although the unetched sample has a thicker Au coverage due to Au dissolution or dynamic movements as discussed for the etched sample.

We conclude that there are no observable oxidized Cr peaks at a higher oxidation state (~576 eV). Thus, there is no formation of Cr oxide that can block any hole carrier transport. We do, however, observe a minor peak at the lower binding energy side of the Cr metallic peak. Currently, we are not able to account for the presence of this peak.

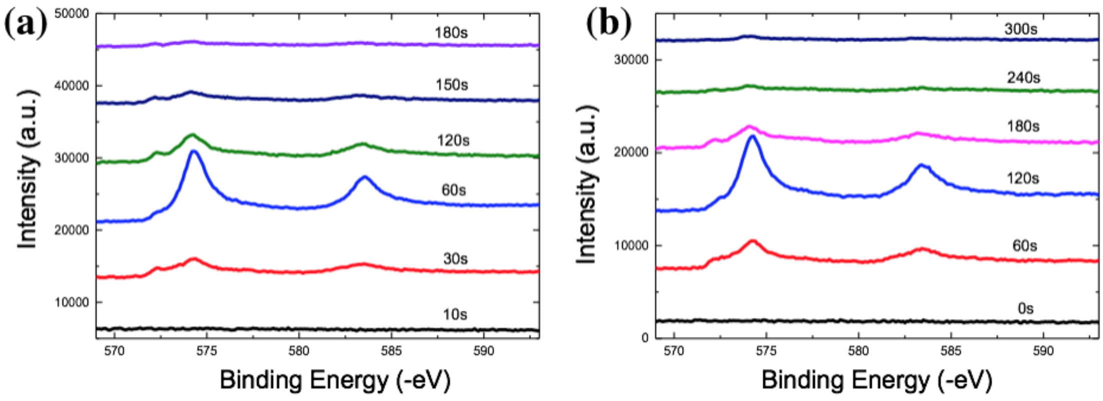

Figure S13 XPS depth profile of tri-layer structure. (a) XPS Cr $2p_{3/2}$ core-level depth profile for 20 mins MacEtched Au/Cr/Au 15/5/10 nm sample, (b) XPS Cr $2p_{3/2}$ core-level depth profile for unetched Au/Cr/Au 15/5/10 nm sample.

S14. Discussions on galvanic displacement reaction

The galvanic displacement reaction is a form of preferential electrochemical corrosion process that occurs between two metals with dissimilar redox potentials.[10] Galvanic displacement reactions have also been used to explain preferential interaction of metal ions in the creation of porous Si nanowires using different metal impurities.[11] Some examples of the reactions are listed as follows:

$$\mathrm{Cr} + \mathrm{Au}^{3+} \rightarrow \mathrm{Cr}^{3+} + \mathrm{Au} \quad (6)$$

$$3\mathrm{Ni} + 2\mathrm{Au}^{3+} \rightarrow 3\mathrm{Ni}^{2+} + 2\mathrm{Au} \quad (7)$$

$$3\mathrm{Ti} + 2\mathrm{Au}^{3+} \rightarrow 3\mathrm{Ti}^{2+} + 2\mathrm{Au} \quad (8)$$

The above reactions are uni-directional since Au is high up in the anodic galvanic index. This means that when the metals are in contact, there is a tendency for the oxidation of metals with a lower anodic galvanic index. The reverse oxidation of Au is not favourable.[9]

S15. Porosity generation during MacEtch of pillars and holes

Cross-section SEM analysis of a 40 nm Au sample after 20 mins MacEtch is shown in Figs. S15a and S15b below. Fig. S15b shows a contrast between the surface and core of nano-pillar indicating a difference in the density. This likely indicates that the pillar consists of a crystalline core while being surrounded by a porous layer, similar to that reported by Geyer *et al.*[12] Unfortunately, it is difficult to clearly observe the presence of any porous Si layer underneath the Au catalyst, that could be too thin to be revealed by SEM clearly. The porosity beneath the metal catalyst however, shows up more clearly in the etching with circular Au disc as shown in Fig. S15c and S15d.

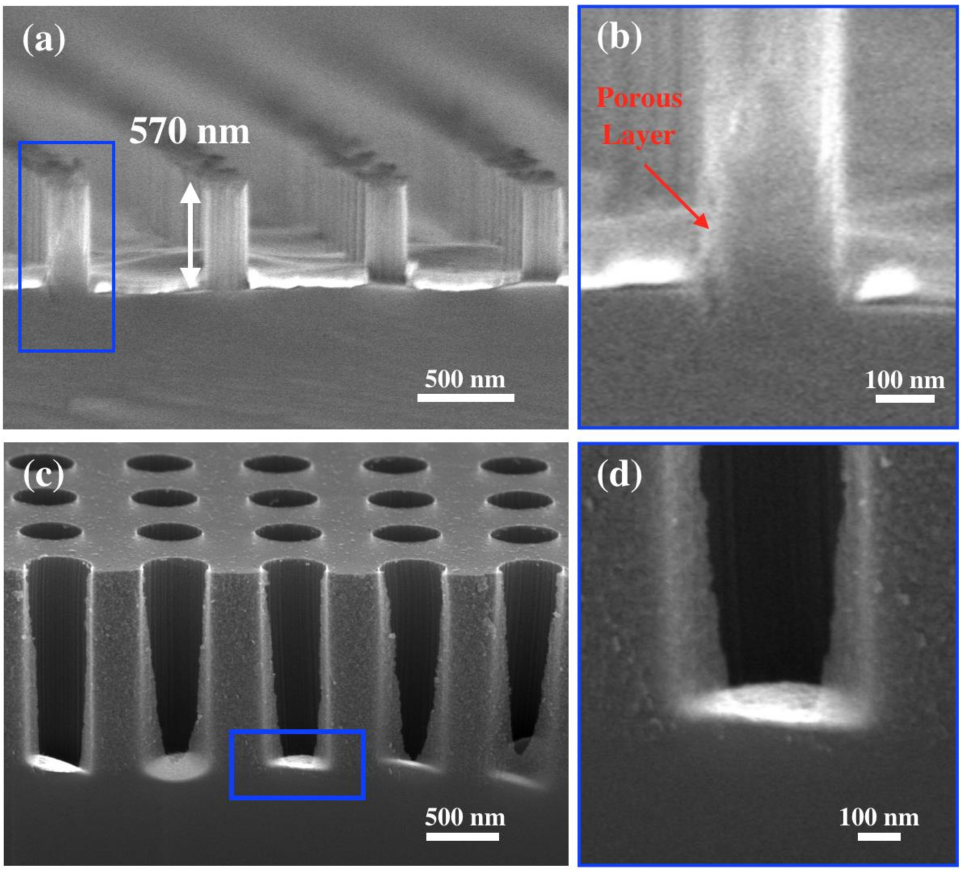

Figure S15 (a) Cross-section SEM micrographs of 40 nm thick Au sample after 20 mins MacEtch, (b) High resolution SEM image of the highlighted nano-pillar in Fig. S15a, (c) MacEtched of nanoholes Au discs catalyst, (d) Zoom-in SEM image of the highlighted region showing presence of a thin porous layer beneath the Au catalyst.

S16. Cross-section view of MacEtched pillars

The cross-section SEM analysis on the 20 nm and 40 nm Au samples after the 20 mins MacEtch is shown in Figs. S16a and S16b, respectively. The average height of these two samples are ~1200 nm and 570 nm. This is close to the expected height of 1175 nm and 550 nm from the 50˚ tilt view in the SEM micrographs as shown in Figs. S16c and S16d, respectively.

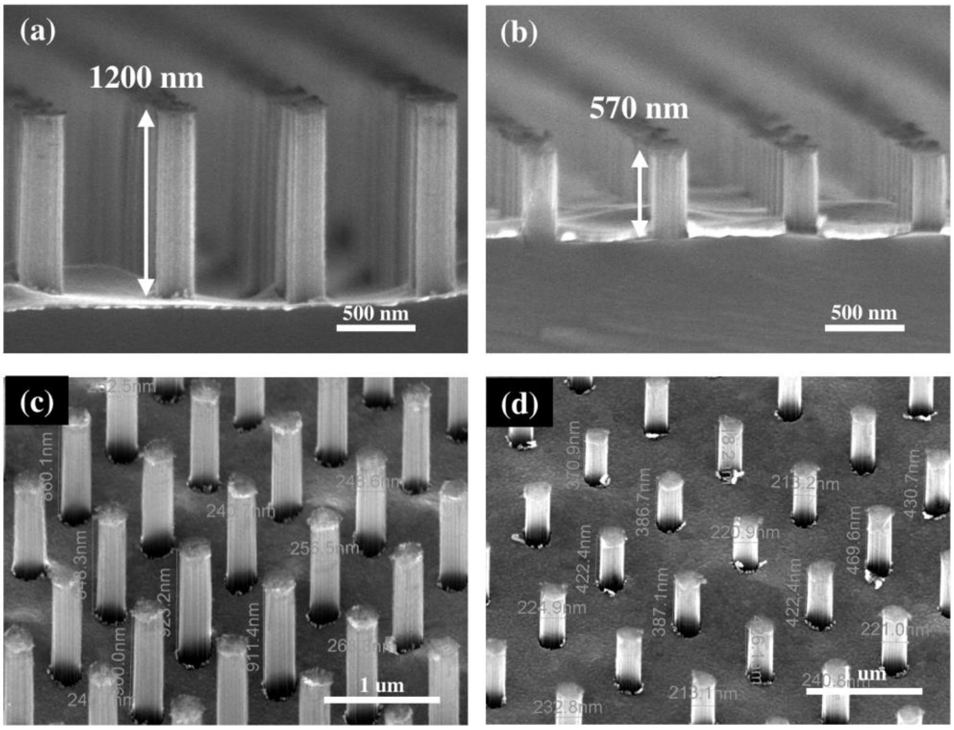

Figure S16 Cross-section SEM micrographs of (a) 20 nm, (b) 40 nm thick Au sample after 20 mins MacEtch. 50˚ tilt view SEM micrograph of (c) 20 nm, (b) 40 nm thick Au sample after 20 mins MacEtch.

S17. References

1 Vogel, N., Goerres, S., Landfester, K. & Weiss, C. K. A convenient method to produce close- and non-closed-packed monolayers using direct assembly at the air-water interface and subsequent plasma-induced size reduction. *Macromol. Chem. Phys.* **212**, 1719-1734 (2011)

2 Li, X. & Bohn, P. W. Metal-assisted chemical etching in HF/H_2O_2 produces porous silicon. *Appl. Phys. Lett.* **77**, 2572 (2000).

3 Peng, K. *et al*. Fabrication of single-crystalline silicon nanowires by scratching a silicon surface with catalytic metal particles. *Adv. Funct. Mater.* **16**, 387-394 (2006).

4 Lee, C. L., Tsujino, K., Kanda, Y., Ikeda, S. & Matsumura, M. Pore formation in silicon by wet etching using micrometer-sized metal particles as catalysts. *J. Mater. Chem.* **18**, 1015-1020 (2008).

5 Peng, K. *et al*. Uniform, axial-orientation alignment of one-dimensional single-crystal silicon nanostructure arrays. *Angew. Chem. Int. Ed.* **44**, 2737-2742 (2005).

6 Peng, K., Lu, A., Zhang, R. & Lee, S. T. Motility of metal nanoparticles in silicon and induced anisotropic silicon etching. *Adv. Funct. Mater.* **18**, 3026-3035 (2008).

7 Toor, F., Oh, J. & Branz, H. M. Efficient nanostructured black silicon solar cell by copper-catalyzed metal-assisted etching. *Prog. Photovolt: Res. Appl.* **23**, 1375-1380 (2015).

8 Oskam, G., Long, J. G., Natarajan, A. & Searson, P. C. Electrochemical deposition of metals onto silicon. *J. Phys. D: Appl. Phys.* **31**, 1927-1949 (1998).

9 To, W. K., Tsang, C. H., Li, H. H. & Huang, Z. Fabrication of n-type mesoporous silicon nanowires by one-step etching. *Nano Lett.* **11**, 5252-5258 (2011).

10 Roberge, P. R. in *Handbook of corrosion engineering*, 331-369 (McGraw-Hill, NY, USA, 2000).

11 Li, X. *et al*. Self-purification model for metal-assisted chemical etching of metallurgical silicon. *Electrochim. Acta* **138**, 476-480 (2014).

12 Geyer, N. *et al*. Model for the mass transport during metal-assisted chemical etching with contiguous metal films as catalysts. *J. Phys. Chem. C* **116**, 13446-13451 (2012).
